# Socioeconomic inequalities in adolescent mental wellbeing in China—a longitudinal path analysis of sport opportunities, exercise self-efficacy, and physical activity

**DOI:** 10.3389/fpubh.2026.1916205

**Published:** 2026-10-20

**Authors:** Nuobei Gongga, Yao Lu, Seongno Lee

**Affiliations:** 1 Department of Physical Education, Hanyang University, Seoul, Republic of Korea; 2 College of Sports, Sejong University, Seoul, Republic of Korea

**Keywords:** adolescent mental health, China, community sport, exercise self-efficacy, longitudinal path analysis, physical activity, school sport, socioeconomic inequality

## Abstract

**Background:**

Mental wellbeing among adolescents in China is of increasing concern. However, previous studies have shown that adolescents from lower socioeconomic backgrounds have fewer opportunities to participate in sport, develop exercise self-efficacy, be physically active, and maintain good mental wellbeing. The temporal trajectories of these variables have not been tested in previous studies.

**Objective:**

This study aimed to determine whether school- and community-based sport opportunities, exercise self-efficacy, and physical activity mediate the relationship between socioeconomic status and adolescents’ mental wellbeing in China.

**Methods:**

Data from three waves of adolescents were used to perform an observed-variable path analysis in R with the lavaan package. At Time 1, 328 adolescents were observed. At Time 2, 294 adolescents were followed to assess variables related to school- and community-based sport opportunities, exercise self-efficacy, and physical activity. At Time 3, 240 adolescents were assessed for their mental wellbeing. Analyses used full-information maximum likelihood methods to handle missing data and 1,000 bootstrap draws to assess the indirect effects of the variables of interest. Because the Time 2 variables were measured concurrently, their hypothesised ordering is theoretical rather than observed, and only the Time 1 to Time 2 to Time 3 structure is longitudinally ordered.

**Results:**

Adolescents of higher socioeconomic status had greater opportunities to participate in sport within their schools (0.443, *p* < 0.001) and within their communities (0.457, *p* < 0.001). Greater opportunities to participate in school and community sports were associated with higher levels of exercise self-efficacy (0.327, *p* < 0.001, 0.339, *p* < 0.001). Higher levels of exercise self-efficacy were associated with greater physical activity (0.354, *p* < 0.001). Higher levels of physical activity were associated with higher mental wellbeing later in adolescence (0.536, *p* < 0.001). Furthermore, the indirect effects of socioeconomic status on mental wellbeing were significant via both the school-based sport pathway (0.029, 95% CI [0.014, 0.050]) and the community-based sport pathway (0.031, 95% CI [0.016, 0.053]), yielding a total indirect effect of 0.061 (95% CI [0.033, 0.096]). Finally, these variables accounted for 76.9% of the variance in mental wellbeing among adolescents at Time 3.

**Conclusion:**

These findings are consistent with the proposition that higher socioeconomic status is associated with greater opportunities to participate in school and community sports, higher levels of exercise self-efficacy, higher rates of physical activity, and better later mental wellbeing. Because the design was observational and the Time 2 mediators were measured concurrently, these findings identify potentially modifiable correlates and plausible intervention targets rather than demonstrated causal mechanisms. With this caveat, policies that aim to increase sport opportunities in schools and communities, improve the accessibility of community sport, and strengthen adolescents’ confidence in engaging in physical activity—particularly for adolescents of lower socioeconomic status or those living in rural communities—may merit consideration.

## 1 Introduction

Adolescent mental health is one of the major public health challenges of the 21st century. Of the estimated 1.1 billion adolescents aged 10–19 worldwide, an estimated one in seven adolescents lives with some form of mental health condition (1). Two of the most common mental health conditions among adolescents are depression and anxiety (1). China is home to the world’s largest adolescent population, with over 250 million adolescents living within its borders (2). Despite the country’s rapid development and growth in recent years, mental health among adolescents in China has become a major concern for the nation’s educators, schools, and mental health officials.

According to the Chinese National Mental Health Development Report for the 2019–2020 academic year, depressive symptoms represent a substantial burden among secondary school students in China (2). More recent estimates from over one million adolescents in China report that 26.17% of children and adolescents experience depressive symptoms (3). Furthermore, research conducted during COVID-19 school closures linked adolescent mental health to family discussion and household context (4). Thus, adolescent mental health in China is not only an individual problem but also a social and environmental issue shaped by the resources available to adolescents within their families, schools, communities, and the country as a whole.

Socioeconomic status (SES) is one of the most important social determinants of an adolescent’s mental wellbeing. Studies of adolescents from families with low socioeconomic status have indicated that these adolescents tend to experience higher levels of psychological distress than adolescents of higher socioeconomic status (5). China is also among the most economically unequal countries in the world, with a household income Gini coefficient of 0.46 or higher for several decades (6, 7). These socioeconomic inequalities may have a profound impact on adolescents’ development in the country, particularly on their access to physical activity. Thus, to understand the impact of socioeconomic status on adolescents’ mental wellbeing in China, it is first necessary to examine the environments in which they live and study.

Physical activity has been shown to reduce the symptoms of depression, anxiety, and psychological distress among adolescents, and to improve the mental wellbeing of those adolescents (8). However, adolescents from lower socioeconomic backgrounds in China are less likely to be physically active than those from higher socioeconomic backgrounds (9). A recent structural equation modelling study of 2,747 children and adolescents in China found that community environment and awareness of sports policies were positively associated with moderate-to-vigorous physical activity (10). Thus, opportunities for adolescents in China to participate in school- or community-based sports may significantly impact their mental wellbeing.

Exercise self-efficacy is a concept that relates to adolescents’ ability to participate in or remain active in physical exercise (11, 12). Access to school and community sport opportunities for adolescents to participate in sports may increase a young adolescent’s exercise self-efficacy, leading to increased physical activity. In contrast, adolescents who lack opportunities to participate in sports may develop low exercise self-efficacy, which can lead to reduced physical activity.

Based on the information presented above, various factors contribute to adolescents’ mental wellbeing in China. The present study uses longitudinal path analysis to examine whether socioeconomic status at Time 1 is associated with mental wellbeing at Time 3 through school and community sport opportunities, exercise self-efficacy, and physical activity at Time 2. The guiding research question was to what extent school and community sport opportunities, exercise self-efficacy, and physical activity mediate the association between SES and later mental wellbeing among Chinese adolescents. Figure 1 presents the conceptual model.

**Figure 1:**
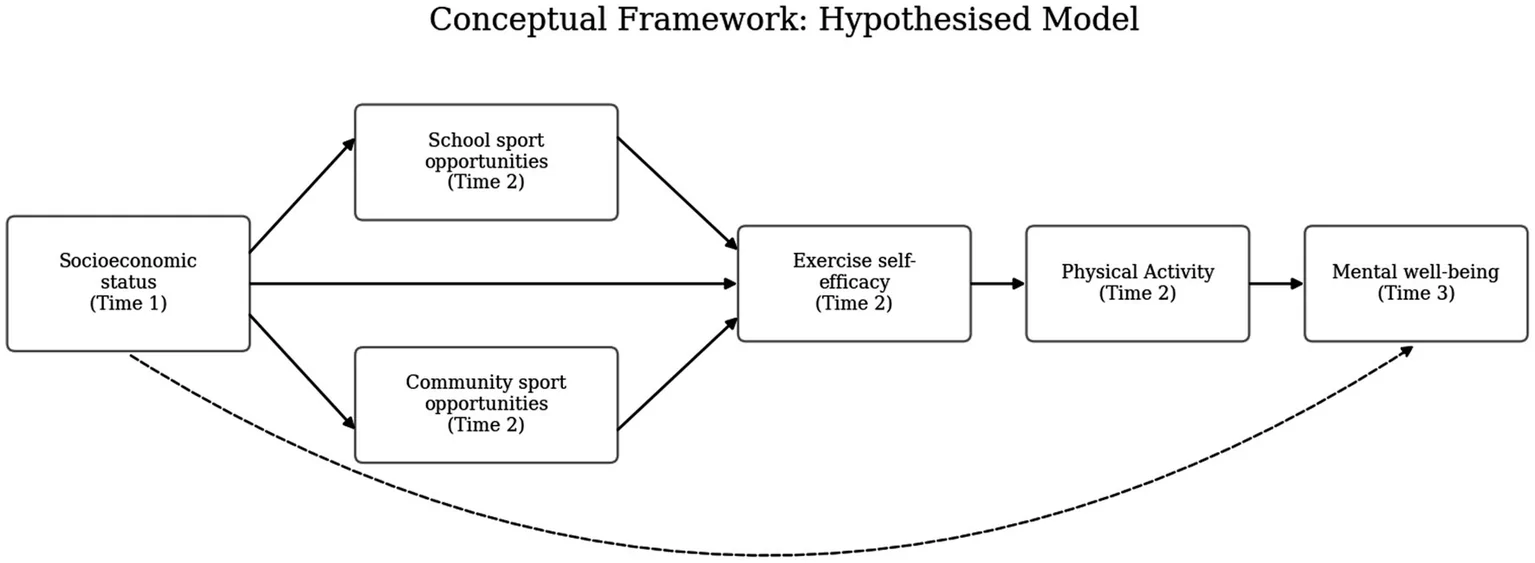
Conceptual framework linking socioeconomic status at Time 1 to mental wellbeing at Time 3 through school and community sport opportunities, exercise self-efficacy, and physical activity. Covariates controlled in all endogenous paths: baseline mental wellbeing, age, sex, rural residence, and grade. Dashed line = residual direct effect of SES (Time 1) on mental wellbeing (Time 3), estimated alongside the indirect pathways shown above.

SES T1 → school sport opportunities T2 and community sport opportunities T2 → exercise self-efficacy T2 → physical activity T2 → mental wellbeing T3. The model also estimated direct paths from SES T1 to exercise self-efficacy T2 and to mental wellbeing T3, and controlled for baseline mental wellbeing, age, sex, rural residence, and grade level. School sport opportunities, community sport opportunities, exercise self-efficacy, and physical activity were all measured at Time 2. Only the Time 1 to Time 2 and Time 2 to Time 3 transitions are longitudinally ordered; the sequence hypothesised among the Time 2 constructs reflects a theoretical expectation drawn from social cognitive theory rather than an observed temporal order (Sections 3.4 and 5.4).

## 2 Literature review

### 2.1 Socioeconomic status and adolescent mental wellbeing in China

The mental wellbeing of adolescents from disadvantaged socioeconomic backgrounds in China has been studied in numerous international and domestic research reports. Such studies indicate that adolescents from disadvantaged socioeconomic backgrounds exhibit higher rates of depressive symptoms, symptoms of anxiety, and stress (3, 13–16). Furthermore, similar longitudinal studies show that the mental health of children and adolescents from disadvantaged socioeconomic backgrounds is enduring over time (17). These findings support the rationale for including a socioeconomic status variable as a predictor of adolescents’ mental wellbeing in this study.

### 2.2 School and community sport opportunities as structural mediators

Research on sport participation and physical activity shows that socioeconomic position shapes the environments in which adolescents can be active. In China, higher family socioeconomic status is associated with higher physical activity among children and adolescents (9), while parental support and parental physical exercise help explain socioeconomic differences in organised sport participation (18). School, family, and community environments also influence adolescent physical activity and fitness, with community environments showing particular relevance for moderate-to-vigorous physical activity (10). Ecological analyses further show that family, school, and community microsystems influence adolescent exercise behaviour (19). These findings justify modelling school and community sport opportunities as structural mediators rather than as background descriptors.

### 2.3 Exercise self-efficacy and physical activity

Exercise self-efficacy is the cognitive mechanism at the centre of Bandura’s social cognitive theory, as it embodies adolescents’ feelings of being able to remain active despite any barriers (11, 12). For adolescents in China, research indicates that exercise self-efficacy acts as the mediator between social support, the sport context, physical activity, and fitness; it is also one of the strongest predictors of physical activity behaviour (5, 20–22). Furthermore, related studies in Hong Kong and mainland China reveal that psychological resilience and mastery in physical activity can influence exercise self-efficacy and related mental health outcomes (20, 23).

Exercise self-efficacy in this study denotes barrier self-efficacy, meaning confidence in exercising despite obstacles, rather than task self-efficacy, meaning confidence in performing particular activities. Two mechanisms plausibly link sport opportunities to it. First, adolescents with fewer opportunities may face more persistent barriers and, through repeated experiences of being unable to exercise as intended, may develop lower confidence in overcoming them. Second, greater access may provide more experience with physical activity and more of the motivational resources needed to sustain it, building confidence in exercising despite barriers. Because socioeconomic status shapes the cumulative resources and experiences available to adolescents over time and precedes both constructs in this design, it may also be directly associated with exercise self-efficacy. Barrier self-efficacy may also relate directly to anxiety, depression, and stress, independent of physical activity.

### 2.4 Physical activity and mental wellbeing

The final link in the framework is the protective role of physical activity for adolescents’ wellbeing. Intervention reviews and studies using structural equation modelling show that adolescents who engage in more physical activity experience fewer depressive symptoms, better mental health, and higher quality of life (8, 24–26). However, surveillance studies show that most Chinese adolescents are inactive (27, 28). Furthermore, because sport environments and socioeconomic status are associated with physical activity among Chinese youth (9, 10), the link between physical activity and mental wellbeing is likely the final behavioural variable to be investigated in relation to the mental wellbeing of Chinese adolescents.

### 2.5 Summary of the literature review and study hypotheses

The existing literature suggests that each of these stages of the proposed model has some relationship to the following stage; that is, that individuals’ socioeconomic status is related to their ability to access certain types of sport environments, which is in turn associated with their exercise self-efficacy, which is associated with their physical activity, which is associated with their mental wellbeing. Few studies have tested these relationships in the same model. The present study therefore proposed that socioeconomic status at Time 1 would be associated with exposure to sport environments and directly with exercise self-efficacy at Time 2; that both sport environment variables would be associated with exercise self-efficacy and physical activity at Time 2; and that physical activity at Time 2 would be associated with mental wellbeing at Time 3, in addition to any direct effect of socioeconomic status on mental wellbeing at Time 3.

## 3 Methods

### 3.1 Design and participants

Three waves of questionnaire data were available for analysis. Data on socioeconomic status and mental wellbeing were collected at Time 1; data on sport opportunity and behavioural mediators were collected at Time 2; and data on mental wellbeing were collected again at Time 3.

Participants were secondary school students recruited from schools across 12 Chinese provinces and municipalities (Beijing, Shanghai, Zhejiang, Jiangsu, Shandong, Henan, Hubei, Hunan, Guangdong, Sichuan, Yunnan, and Guizhou), drawn from both public (68.9%) and private (31.1%) schools and from both urban/suburban (59.5%) and rural (40.5%) settings. Individual school identifiers were not retained in the analytic dataset, so the exact number of participating schools cannot be reported and school-level clustering could not be quantified or adjusted for statistically; this is addressed as a limitation in Section 5.6. Recruitment followed a multi-site convenience sampling approach rather than a probability-based national sample; consequently, while the sample spans a broad geographic range, it should not be assumed to be nationally representative of Chinese adolescents, and inferences are best restricted to adolescents in similar school settings within the sampled regions. Data were collected across three survey waves in September 2025, January 2026, and June 2026, respectively, with an overall follow-up period of approximately nine months.

The longitudinal questionnaire was used to assess the relationships among socioeconomic status, sport opportunities, and mental wellbeing in adolescents. At Time 1, 328 adolescents were surveyed. At Time 2, 294 adolescents were surveyed. At Time 3, 240 adolescents were surveyed. Because not all adolescents participated in all three questionnaire waves, the data are unequal. However, rather than deleting adolescents with incomplete records, the researchers used full-information maximum likelihood (FIML) estimation. FIML does not impute or estimate replacement values for individual missing responses; instead, it uses all available observed data directly in the estimation of model parameters, under the assumption that data are missing at random conditional on the variables in the model.

The data were balanced by adolescent sex and school level. At Time 1, the average age of the adolescents was 14.36 years (SD = 1.48). Just over half of the adolescents were female (51.8%), and the remainder were male (48.2%). Approximately 40.5% of adolescents lived in rural areas, while the remainder lived in urban or suburban areas. Approximately 48.8% of the adolescents were attending senior secondary schools within the study. About 31.1% of the adolescents attended private schools, while the remainder attended public schools. Additionally, 19.5% of the adolescents did not live with both parents. The retention rate from Time 1 to Time 2 was 89.6%, while that from Time 1 to Time 3 was 73.2%. These retention rates are broadly comparable to those reported in similar school-based longitudinal studies; a formal attrition analysis comparing retained and non-retained participants on baseline characteristics is reported in Section 4.1.

### 3.2 Measures

Socioeconomic status (at Time 1) was measured using a composite of six components rated on a common 1–5 metric, namely father’s education, mother’s education, family economic situation, ability to pay for school needs, ability to afford sport/exercise costs, and a household-assets index derived from seven binary ownership items (smartphone, computer, home internet access, a private study room, a bicycle, a car, and sports equipment) and rescaled to a 1–5 range based on the proportion of assets owned. Item scores were averaged to form the composite, with higher scores indicating higher socioeconomic status. At Time 1, the mean socioeconomic status score was 3.04 on a 1–5 scale, with a standard deviation of 1.06.

Because SES combined largely independent socioeconomic resources (parental education, household economic circumstances, affordability, and material assets) rather than repeated indicators of a single underlying trait, it is conceptually a formative rather than a reflective composite. Cronbach’s alpha is nonetheless reported below for descriptive comparability with the other, reflectively measured scales, and should not be interpreted as evidence that the SES items form a unidimensional construct.

School sport opportunities (Time 2) were measured with 10 items covering access to facilities, equipment, regular physical education lessons, school encouragement of sport, extracurricular sport, teacher support, inclusion of students from all backgrounds, affordability, safety of facilities, and equal opportunity for boys and girls to participate. Community sport opportunities (Time 2) were measured with a parallel set of 10 items covering access to nearby facilities and parks, ease of reaching them, neighbourhood safety, affordability, organised community sport programmes, availability of open spaces, ease of access, parental permission to use facilities, and accessibility for lower-income families. Each item was rated on a 5-point scale (1 = lowest level of access or agreement, 5 = highest) and item scores were averaged to form each composite, with higher scores indicating greater opportunity. At Time 2, mean scores indicated that students had access to 2.96 school sport opportunities (SD = 1.18) and 3.02 community sport opportunities (SD = 1.17).

Exercise self-efficacy (Time 2), conceptualised as barrier self-efficacy (Section 2.3), was measured with 8 items asking participants to rate their confidence that they could exercise when tired, when busy with schoolwork, without friends to exercise with, on a weekly basis, when learning new sports, when not skilled at a sport, when finding time was difficult, and without expensive equipment, each on a 1–5 scale, averaged to form the composite. Physical activity (Time 2) was measured with 5 items, four rated on a 1–5 scale (frequency of physical education lessons, after-school sport, weekend exercise, and participation in organised sport clubs or teams) and one assessing the number of days in the past week (0–7) on which the adolescent was active for at least 60 min; all five items were averaged into a single composite. Because this composite blends a 0–7 day-count item with four 1–5 items, its mean (e.g., 2.99 at Time 2) is an index score, not a count of minutes of activity. Mental wellbeing (Time 1 and Time 3) was measured with the same 15 items and scoring procedure at both waves, namely 10 positively worded wellbeing items (e.g., feeling cheerful, calm, hopeful, supported) scored directly, and 5 negatively worded symptom items (e.g., feeling sad, anxious, lonely, stressed, unable to enjoy things) reverse-scored, all on a 1–5 scale and averaged, with higher scores indicating better mental wellbeing. At Time 3, mental wellbeing scores indicated a mean of 2.97 (SD = 1.15).

All measures were developed for the present study from the constructs identified in the literature review. None reproduces a published scale in full, and no expert panel review or confirmatory factor analysis was conducted before use. The composites are therefore study-specific indices rather than validated instruments, as discussed in Section 5.6. Full item content is provided in Supplementary file S2.

### 3.3 Reliability and data screening

The internal consistency of the reflectively measured scales was assessed using Cronbach’s alpha. The value for school sport opportunity was above 0.96. The value for community sport opportunity was above 0.96. The value for exercise self-efficacy was above 0.95. The value for physical activity was above 0.93. The value for measuring mental wellbeing was above 0.97. Alpha for the SES composite (above 0.92) is reported for descriptive comparability only, because SES combines formative indicators (Section 3.2) and the coefficient should not be read as evidence of unidimensionality. For the reflectively measured constructs, these high coefficients indicate that the items within each scale were measuring the same construct. Exploratory factor analyses (Supplementary file S2) supported a single factor for every composite, with parallel analysis retaining one factor in each case (first eigenvalues 4.00 to 11.64; loadings 0.67 to 0.90). This supports combining the items but does not establish construct validity. While it is possible that the high values for these coefficients were partly attributable to the similarity of wording among items within each construct, the results nevertheless indicate that the constructs as a whole were reliably measured.

The descriptive statistics for each model variable were examined prior to estimating the path model. Each variable was scored on a scale of 1 to 5 for perception or experience. The means for each variable were clustered around the midpoint of the scale. The correlations between the variables were positive and generally consistent with the hypothesised model. For example, socio-economic status was significantly positively correlated with school sport opportunities at Time 2, community sport opportunities at Time 2, physical activity at Time 2, and mental wellbeing at Time 3. Additionally, as expected, there was a strong correlation between the physical activity variable at Time 2 and mental wellbeing at Time 3.

### 3.4 Questionnaire development and wave timing

The questionnaire was developed from the conceptual pathway described in the literature review and divided into separate sections to assess the various aspects of the proposed study. Each adolescent was also assigned the same code within the questionnaire to allow them to be matched to their responses across the questionnaire while maintaining their anonymity in the data. Three waves of the questionnaire were developed to minimise bias from measuring the same adolescents at the same time. Additionally, the variables assessed within each wave of the questionnaire were correlated with the concept of the direction of impact each variable was expected to have on the following variables. Thus, variables such as SES and mental wellbeing were assessed at Wave 1 to account for their status prior to the subsequent (Wave 2 and Wave 3) waves of data collection; this is an observational longitudinal study, and no intervention was administered to participants. Variables related to sport opportunities, self-efficacy, and physical activity were assessed at Wave 2. Finally, the variable for mental wellbeing was assessed at Wave 3 to determine whether the intermediate variables were associated with adolescents’ mental wellbeing at the end of the study period.

School sport opportunities, community sport opportunities, exercise self-efficacy, and physical activity were all collected within the same Wave 2 questionnaire. Only the structure from SES (Wave 1, which precedes every Wave 2 construct including exercise self-efficacy) to the Wave 2 variables to mental wellbeing (Wave 3) is therefore longitudinally ordered. The sequence hypothesised among the Wave 2 constructs is theoretically motivated, drawing on social cognitive theory, rather than empirically demonstrated, and the data cannot rule out alternative or reciprocal orderings, for example self-efficacy shaping perceived sport opportunities. The model does include a partially competing specification, with direct paths from both types of sport opportunity to physical activity that bypass self-efficacy alongside the self-efficacy route (Section 4.4 and Figure 2). Both routes were significant. Testing further orderings would require waves that separate these constructs in time. The Wave 2 sequence is therefore described throughout as a prospective pathway rather than a demonstrated mechanism.

**Figure 2:**
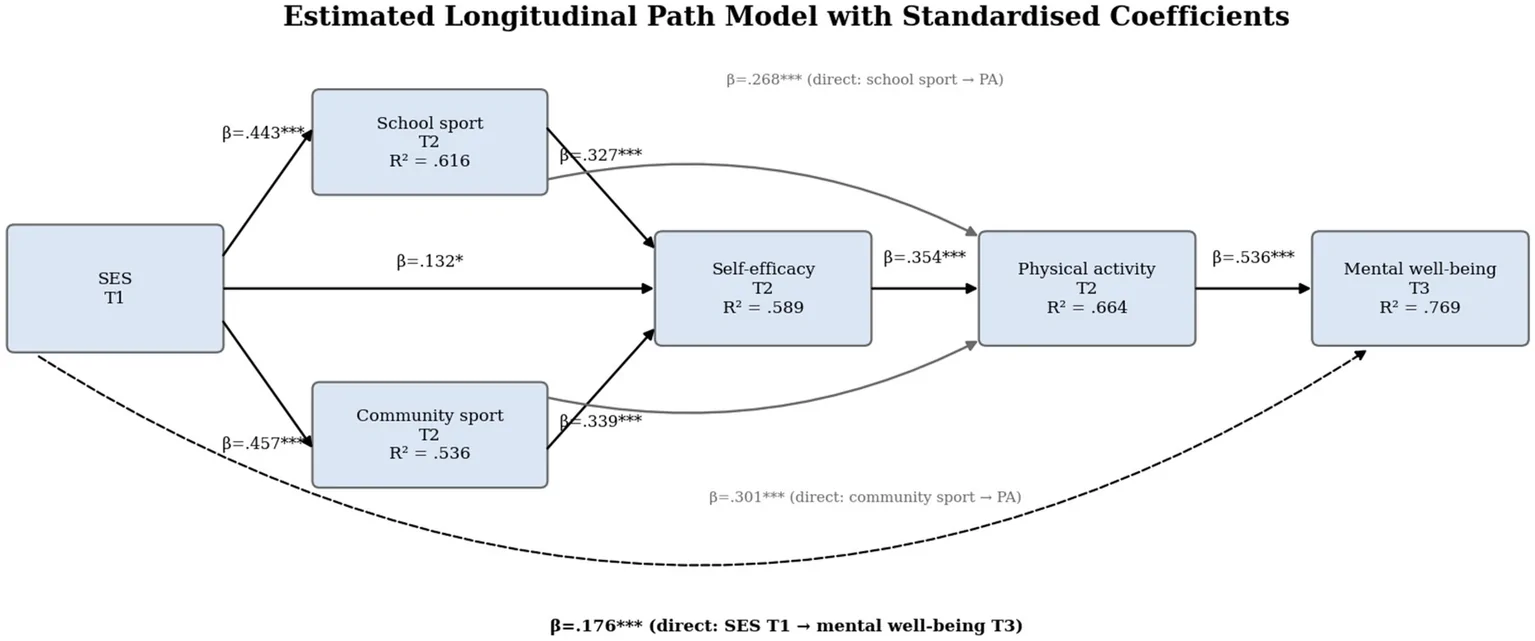
Estimated longitudinal path model with standardised coefficients and explained variance (*R*^2^) for each endogenous construct. Covariates and the non-significant direct path from SES to physical activity are omitted for readability. ^*^p < 0.05, ^***^p < 0.001. Solid black arrows show the hypothesised pathways; grey curved arrows show additional direct paths from sport opportunities to physical activity; the dashed arc shows the residual direct effect of SES on Time 3 mental wellbeing. All paths were estimated simultaneously, adjusting for baseline mental wellbeing, age, sex, rural residence, and grade (not shown). The direct path from SES to physical activity (*β* = −0.040, *p* = 0.471) is not shown.

### 3.5 Statistical analysis

The analysis was performed in R (31) using the lavaan package (32). The lavaan package uses maximum likelihood estimation with full information maximum likelihood for missing data and bootstrap standard errors. Specifically, 1,000 bootstrap draws were requested, and all 1,000 bootstrap draws were successful. There were 328 participants in the model and 74 estimated model parameters. The missing-data structure comprised three distinct patterns, namely complete data across all three waves (*n* = 240), Time 1 and Time 2 data but not Time 3 (*n* = 54), and Time 1 data only (*n* = 34). FIML used all available data from all 328 participants to estimate the model parameters; no participants were deleted. The main predictors in the model included variables for socioeconomic status at Time 1, sport opportunities in school at Time 2, sport opportunities in the community at Time 2, exercise self-efficacy at Time 2, physical activity at Time 2, and mental wellbeing at Time 3. Additionally, variables for baseline levels of mental wellbeing, age, sex, rural residence, and grade level were included as covariates.

The path model included effects from socioeconomic status to opportunities to participate in school and community sports, to self-efficacy in physical exercise, and to physical exercise, effects from opportunities to participate in school or community sports to self-efficacy in physical exercise, effects from self-efficacy in physical exercise and opportunities to participate in school or community sports to physical exercise, and effects from physical exercise and socioeconomic status to mental wellbeing at Time 3. Additionally, a covariance was estimated between opportunities to participate in school and community sports, as these variables are related. Furthermore, bootstrapped indirect and total effects of socioeconomic status on mental wellbeing, operating through school and community sport opportunities, exercise self-efficacy, and physical activity, were calculated. Unless otherwise noted, indirect and total effects are reported throughout in the unstandardised (raw) metric, consistent with standard practice for bootstrapped effects in lavaan; standardised path coefficients are reported separately for the direct paths. The model fit was examined using measures of the chi-square statistic, the comparative fit index (CFI), the Tucker-Lewis index (TLI), the root mean square error of approximation (RMSEA), and the standardised root mean square residual (SRMR). Because this model had very low degrees of freedom, the interpretation of the RMSEA and the TLI was performed with particular caution, as these indices are known to behave unpredictably in low-df models; this is discussed further in Section 5.4. The complete lavaan model syntax used to produce these results is provided in Supplementary file S1 to support transparency and replication.

## 4 Results

### 4.1 Participant retention and descriptive patterns

The longitudinal sample exhibited the desired unequal wave structure. Time 1 included 328 adolescents, Time 2 included 294 adolescents, and Time 3 included 240 adolescents. Thus, 34 adolescents were lost between Time 1 and Time 2, and 54 between Time 2 and Time 3. Thus, 240 adolescents participated in all three waves of the study, and 294 participated in the first two waves. The FIML method enabled the analysis to use all available data to estimate the model’s parameters.

The descriptive statistics for the major constructs all exhibited values within the expected ranges on the 1 to 5 scale. For instance, adolescents at Time 1 had a mean score of 3.04 (SD = 1.06) on the SES scale and a mean score of 3.00 (SD = 1.13) on the mental wellbeing scale. At Time 2, adolescents had a mean score of 2.96 (SD = 1.18) on the availability of school sport opportunities, 3.02 (SD = 1.17) for community sport opportunities, 2.99 (SD = 1.15) for their level of exercise self-efficacy, and 2.99 (SD = 1.07) on the physical activity composite index (this index combines four 1–5 frequency items with a 0–7 day-count item, as described in Section 3.2, and does not represent minutes of activity). Finally, adolescents at Time 3 had a mean score of 2.97 (SD = 1.15) on the mental wellbeing scale.

#### 4.1.1 Attrition analysis

To evaluate the plausibility of the missing-at-random assumption underlying FIML, participants retained through Time 3 (*n* = 240) were compared with those not retained (*n* = 88, comprising the 34 participants with Time 1 data only and the 54 with Time 1 and Time 2 data only) on baseline characteristics. Retained and non-retained participants did not differ significantly on baseline SES (*M* = 3.01 vs. 3.11, *t* = −0.78, *p* = 0.437), baseline mental wellbeing (*M* = 2.97 vs. 3.08, *t* = −0.78, *p* = 0.436), age (*M* = 14.38 vs. 14.30, *t* = 0.45, *p* = 0.653), sex (51.3% vs. 53.4% female, *p* = 0.824), rural residence (43.3% vs. 33.0% rural, *p* = 0.117), grade level (*p* = 0.545), school type (*p* = 0.399), or living arrangement (*p* = 1.00). While this comparison cannot rule out attrition related to unmeasured variables, the absence of significant differences on these observed baseline characteristics lends some support to the plausibility of a missing-at-random mechanism.

### 4.2 Model fit

Model convergence was typically achieved after 123 iterations. The chi-square test was significant, chi-square(3) = 36.98, *p* < 0.001, indicating that the model’s exact fit was not supported. The CFI (0.976) and SRMR (0.014) were favourable, but the TLI was low at 0.686 and the RMSEA was high at 0.186 (90% CI [0.135, 0.242]), both indicating poor fit by conventional standards. Considered together, these indices present a mixed picture, and the model should not be described as providing a good overall fit to the data; rather, incremental fit relative to the (very poorly fitting) baseline model was favourable, while absolute fit was not. Because the model had only three degrees of freedom, both the RMSEA and the TLI are known to behave unpredictably and can be sensitive to minor, substantively unimportant deviations from the null hypothesis; this low-df sensitivity is a plausible partial explanation for the poor absolute-fit indices, but it does not fully resolve the discrepancy, and readers should interpret the model as a useful but imperfect and only partially confirmed representation of the data, alongside the R-square values and pattern of substantive path coefficients reported below.

### 4.3 Pathway from socioeconomic status to sport opportunities

Socioeconomic status at Time 1 significantly predicted both school- and community-based sport opportunities at Time 2. The path from socioeconomic status to school sport opportunities was positive and significant (unstandardised estimate = 0.493, *p* < 0.001; standardised beta = 0.443). The path from socioeconomic status to community sport opportunities was also positive and significant (unstandardised estimate = 0.506, *p* < 0.001; standardised beta = 0.457). Adolescents with higher socioeconomic status at Time 1 had greater access to school and community sport at Time 2.

Mental wellbeing at Time 1 was also significantly related to school sport opportunities at Time 2 (standardised beta = 0.199, *p* < 0.001), but it did not significantly relate to community sport opportunities. Adolescents who lived in rural areas had significantly fewer opportunities to access both school (standardised beta = −0.377, *p* < 0.001) and community sport opportunities (standardised beta = −0.408, *p* < 0.001). However, age, sex, and grade level were not significantly associated with access to sport opportunities. Overall, sports opportunity inequality is both socioeconomic and spatial.

### 4.4 Pathway from sport opportunities to exercise self-efficacy and physical activity

Both school and community sport opportunities were significantly predictive of exercise self-efficacy at Time 2. School sport opportunities were significantly associated with exercise self-efficacy at Time 2, *b* = 0.321, *p* < 0.001, and a standardised beta of 0.327. Community sport opportunities also had a significant effect on exercise self-efficacy at Time 2, with an unstandardised estimate of 0.334 (*p* < 0.001) and a standardised beta of 0.339. Socioeconomic status was also significantly associated with exercise self-efficacy, though with a smaller effect; its unstandardised estimate was 0.144 (*p* = 0.025), and its standardised beta was 0.132. These results indicate that one mechanism through which socioeconomic advantage is associated with higher levels of exercise self-efficacy is the opportunity to participate in school- and community-based sports.

At Time 2, physical activity was best predicted by three main variables. First, exercise self-efficacy was significantly associated with physical activity, with an unstandardised estimate of 0.328 (*p* < 0.001) and a standardised beta of 0.354. Second, students’ opportunities to participate in school-based sports were also significantly associated with physical activity; school sport opportunities had an unstandardised estimate of 0.244 (*p* < 0.001) and a standardised beta of 0.268. Third, participation in community-based sports was also significantly associated with physical activity; community sport opportunities had an unstandardised estimate of 0.276 (*p* < 0.001) and a standardised beta of 0.301. Finally, although socioeconomic status was associated with physical activity when considered on its own, no significant direct effect of socioeconomic status on physical activity remained once sport opportunities, self-efficacy, mental wellbeing, and the demographic covariates were included in the same equation. Thus, these results are consistent with the association between socioeconomic status and physical activity operating indirectly, through sport-related opportunities and exercise self-efficacy, rather than through a residual direct effect of SES on physical activity itself.

### 4.5 Pathway from physical activity to mental wellbeing

The only variable that was statistically significant in predicting mental wellbeing at Time 3 was physical activity. The unstandardised estimate for the effect of Time 2 physical activity on Time 3 mental wellbeing was 0.567 (*p* < 0.001); the standardised beta was 0.536. Baseline mental wellbeing was also significantly predictive of Time 3 mental wellbeing. The unstandardised estimate was 0.249 (*p* < 0.001), and the standardised beta was 0.249.

However, SES still had a significant direct effect on Time 3 mental wellbeing, even after accounting for the mediating effect of mental wellbeing. The unstandardised direct effect was 0.188 (*p* < 0.001), and the standardised beta was 0.176. The variable for residence in rural areas was significantly negatively associated with mental wellbeing at Time 3. The unstandardised estimate was −0.287 (*p* = 0.003), and the standardised beta was −0.125. Age and sex were not significantly predictive of mental wellbeing at Time 3. However, grade level approached significance in predicting mental wellbeing at Time 3; albeit with a negative direct effect, adolescents in senior grades had lower levels of mental wellbeing than those in junior grades.

### 4.6 Indirect effects and explained variance

The bootstrapped indirect effects (reported here in the unstandardised metric; see Section 3.5) are statistically significant and are consistent with the hypothesised sequential pathway. The indirect effect from SES to sport opportunities, exercise self-efficacy, physical activity, and mental wellbeing was significant, *b* = 0.029, *p* = 0.001 (95% CI [0.014, 0.050]). The indirect effect from SES to these outcomes via community sport opportunities was also significant, *b* = 0.031, *p* = 0.001 (95% CI [0.016, 0.053]). The total indirect effect was also significant, 0.061, *p* < 0.001 (95% CI [0.033, 0.096]). The total effect of SES on Time 3 mental wellbeing was significant, 0.249, *p* < 0.001 (95% CI [0.152, 0.348]).

The model explained a large proportion of the variance in each endogenous variable. For school sport opportunities, R-square was 0.616. For community sport opportunities, R-square was 0.536. The model accounted for 58.9% of the variance in exercise self-efficacy, 66.4% of the variance in physical activity, and 76.9% of the variance in Time 3 mental wellbeing. Thus, the model accounted for a large portion of the variation in the four outcomes of interest.

### 4.7 Summary of the empirical pathway

Figures 2, 3 visually represent the fitted model. Figure 2 presents the standardised path diagram for the model, while Figure 3 displays the indirect-effect figure for the model, which shows that both the school and community sport pathways accounted for statistically significant portions of the effect of socioeconomic status on mental wellbeing later in life.

**Figure 3:**
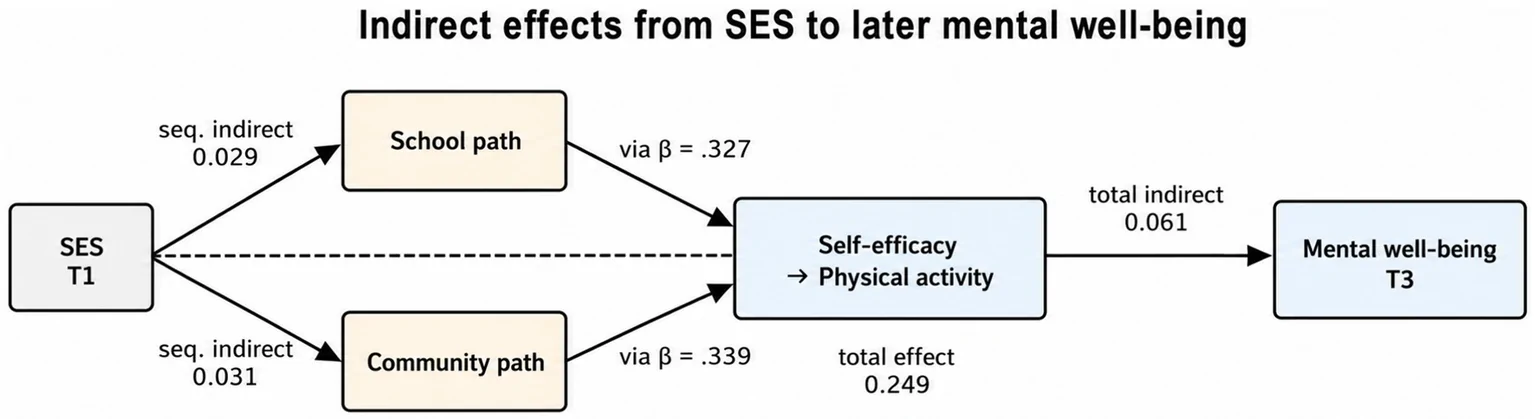
Indirect effects from socioeconomic status to Time 3 mental wellbeing through the school and community sport pathways. Unstandardised estimated from 1,000 bootstrap draws: school sequential path 0.029 [0.014, 0.050]; community sequential path 0.031 [0.016, 0.053]; total indirect effect 0.061 [0.033, 0.096].

The results of the model are consistent with the hypothesised model; that is, opportunities to participate in sport and physically active behaviours were associated with differences in mental wellbeing between adolescents of different socioeconomic statuses. The early pathways into physical activity and sport opportunities were strongest from socioeconomic status to opportunities to participate in school and community sport activities. However, the strongest relationship within the model existed between physical activity and mental wellbeing at Time 3. Furthermore, although each indirect effect was individually significant, both school and community sport activities contributed to the relationship between socioeconomic status and mental wellbeing.

However, these results only indicate a partial mediation of the relationship between socioeconomic status and mental wellbeing. For instance, even after including these sport-opportunity variables, socioeconomic status remained significantly correlated with mental wellbeing, as did the disadvantages faced by individuals living in rural regions of the country and by those with limited opportunities to participate in sports. Thus, while including sport opportunities as a variable may help reduce differences in mental wellbeing between individuals of different socioeconomic statuses, it will not eliminate all the effects of those socioeconomic differences on adolescents’ mental health (Tables 1–4).

**Table 1:** Participant retention and wave structure.

Wave	*N*	Retention from Time 1	Main variables measured
Time 1	328	100.0%	SES, baseline mental wellbeing, age, sex, residence, grade
Time 2	294	89.6%	School sport opportunities, community sport opportunities, exercise self-efficacy, physical activity
Time 3	240	73.2%	Mental wellbeing

**Table 2:** Model fit indices for the longitudinal path model.

Index	Value	Interpretation
Chi-square	36.980, *df* = 3, *p* < 0.001	Exact fit not supported
CFI	0.976	Good comparative fit
TLI	0.686	Below conventional threshold
RMSEA	0.186, 90% CI [0.135, 0.242]	Poor absolute fit; interpreted cautiously due to low df
SRMR	0.014	Good residual fit

**Table 3:** Main standardised longitudinal path coefficients.

Path	Unstandardised estimate	Standardised beta	*p*-value
SES T1 → School sport opportunities T2	0.493	0.443	<0.001
SES T1 → Community sport opportunities T2	0.506	0.457	<0.001
School sport opportunities T2 → Exercise self-efficacy T2	0.321	0.327	<0.001
Community sport opportunities T2 → Exercise self-efficacy T2	0.334	0.339	<0.001
SES T1 → Exercise self-efficacy T2	0.144	0.132	0.025
Exercise self-efficacy T2 → Physical activity T2	0.328	0.354	<0.001
School sport opportunities T2 → Physical activity T2	0.244	0.268	<0.001
Community sport opportunities T2 → Physical activity T2	0.276	0.301	<0.001
Physical activity T2 → Mental wellbeing T3	0.567	0.536	<0.001
SES T1 → Mental wellbeing T3	0.188	0.176	<0.001
Rural residence → Mental wellbeing T3	−0.287	−0.125	0.003

All values in the “Unstandardised estimate” and “Standardised beta” columns retain their original sign; a negative sign on either value indicates an inverse association. All coefficients shown are significant at *p* < 0.05.

**Table 4:** Indirect effects and explained variance.

Effect or outcome	Estimate/R-square	95% CI or interpretation
SES → school sport → self-efficacy → PA → MWB	0.029	0.014–0.050
SES → community sport → self-efficacy → PA → MWB	0.031	0.016–0.053
Total indirect effect	0.061	0.033–0.096
Total effect of SES on MWB T3	0.249	0.152–0.348
R-square (School sport opportunities T2)	0.616	61.6% of variance explained
R-square (Community sport opportunities T2)	0.536	53.6% of variance explained
R-square (Exercise self-efficacy T2)	0.589	58.9% of variance explained
R-square (Physical activity T2)	0.664	66.4% of variance explained
R-square (Mental wellbeing T3)	0.769	76.9% of variance explained

The indirect- and total-effect estimates in this table are unstandardised (raw-metric) bootstrapped estimates with 1,000 draws; R-square values are unit-free proportions of variance explained and are not on the same scale as the indirect-effect estimates.

## 5 Discussion

### 5.1 Principal findings

The longitudinal path analysis presented in this paper examines the extent to which socioeconomic inequalities in adolescent mental wellbeing in China are associated with inequalities in school and community sport opportunities, exercise self-efficacy, and physical activity. The results generally support the proposed model of mental wellbeing. Students of higher socioeconomic statuses had better access to opportunities to participate in school and community sports; those with better access to such sports had higher levels of exercise self-efficacy; those with higher levels of exercise self-efficacy were more likely to exhibit higher levels of physical activity; and those who exhibited higher levels of physical activity reported higher levels of mental wellbeing. Furthermore, the indirect effect of socioeconomic status on mental wellbeing through school and community sport opportunities was statistically significant.

However, the effect of socioeconomic status on mental wellbeing remained significant even after including variables related to sport opportunities, exercise self-efficacy, physical activity, mental wellbeing at Time 1, and several demographic variables of the participants. Thus, while sport opportunities were associated with mental wellbeing in the population, sport-based factors were only one of many contributing factors to socioeconomic differences in adolescents’ mental wellbeing in China. Other potential factors contributing to adolescents’ mental wellbeing in these groups included family-related issues, such as stress, resources, time, and factors affecting mental wellbeing within those families. However, the significant relationship between mental wellbeing found for the sport-based model suggests a potential intervention to improve adolescents’ mental wellbeing in schools and communities across the country.

### 5.2 Interpretation through social cognitive and ecological theory

The findings align with social cognitive theory, as exercise self-efficacy served as the main cognitive component in the relationship between environmental opportunity and behaviour (11, 12). Opportunities to participate in sports within the school and community can increase self-efficacy, but the lack of such opportunities may lead adolescents to feel less motivated to engage in physical activity. Self-efficacy was, therefore, found to be a significant predictor of physical activity levels within the population, even after the effects of school and community sports access were accounted for in the model. Socioeconomic status was also directly associated with exercise self-efficacy beyond its association with sport opportunities, consistent with an accumulation of resources and experiences over time.

The findings also align with ecological systems theory, in that access to sport opportunities within the environment was associated with an adolescent’s socioeconomic status and with whether they lived in a rural or urban area (19). For instance, adolescents with higher socioeconomic status had better access to both school and community sports than those with lower socioeconomic status. Additionally, adolescents living in rural areas had less access to both schools and community sports than those living in urban areas. Thus, even if adolescents have the motivation to engage in physical activity, their socioeconomic status, rural living, or other environmental factors may create barriers to participation.

### 5.3 Comparison with previous evidence

The findings of this study are consistent with existing research that demonstrates a link between socioeconomic status and physical activity levels among children and adolescents in China (9). Additionally, previous studies have demonstrated the relationships among school, family, and community influences on adolescents’ physical activity and fitness (10). However, this study used a longitudinal modelling approach to examine the links between socioeconomic status at Time 1, sport opportunity and self-efficacy at Time 2, and mental wellbeing at Time 3. While these findings suggest links between these variables, they do not provide evidence of causation due to the research study design.

The study’s findings also generally support previous research on exercise self-efficacy. For instance, Ren et al. reported that exercise self-efficacy mediated the relationship between social support and physical activity among adolescents in China (21). Furthermore, studies based on the social cognitive theory of physical activity suggest that exercise self-efficacy is one of the strongest predictors of physical activity among young individuals (5). The findings of this study are consistent with self-efficacy being associated with both school and community access to sport opportunities, and with such self-efficacy being, in turn, associated with adolescents’ physical activity levels. Because self-efficacy and physical activity were both measured at Time 2, this last association is concurrent rather than prospective, and should not be read as evidence that self-efficacy precedes physical activity in time (Sections 3.4 and 5.4). Barrier self-efficacy may also relate directly to mental wellbeing. However, the model was specified to test a behavioural pathway through physical activity and did not estimate a direct path from exercise self-efficacy to mental wellbeing, so this route remains untested and is a priority for future research.

The relationship between physical activity and mental wellbeing later in adolescence aligns with previous research on the link between physical activity and psychological outcomes among adolescents. Studies report that physical activity interventions can be effective in reducing depressive symptoms among children and adolescents (8). Furthermore, studies among adolescents in China have specifically reported relationships between physical activity and outcomes related to mental wellbeing, including resilience, emotional wellbeing, and general wellbeing (23–25). Thus, these findings support the suggestion that the relationship between physical activity and mental wellbeing can help establish the link between the sport environment accessible to adolescents and their mental wellbeing over time.

### 5.4 Interpretation of model fit

The model fit results require a cautious, balanced interpretation. The CFI and SRMR were favourable, but the low TLI and high RMSEA indicate that the model did not achieve good absolute fit by conventional standards, and the model should not be described as fitting the data well overall. Low degrees of freedom can make the RMSEA and TLI unstable, which may partly explain these results, but this explanation should not be used to dismiss the poor absolute-fit indices outright. More specifically, the high R-square values and the pattern of statistically significant paths that correspond to theoretically expected relationships indicate that the model is a useful, but only partially confirmed and imperfect, representation of the data. Future studies should include additional variables and data points (such as school or community identifiers, to allow multilevel modelling) and, where possible, additional measurement waves that separate the Time 2 constructs (school and community sport opportunities, exercise self-efficacy, and physical activity) in time, so that their hypothesised sequence can be tested directly rather than assumed; as discussed in Section 3.4, the present cross-sectional-within-wave measurement of these constructs means the model tests a plausible, theoretically motivated ordering rather than a directly observed one, and this should be regarded as a design limitation distinct from the fit-index results discussed above.

When interpreting these results, it is important to consider the study’s title. The study’s framework is ecological in structure, but it is not a model that tests for multilevel relationships. While the study is longitudinal and focuses on the pathways of the variables of interest, it did not account for the random effects of schools or communities among the adolescents observed. Thus, the study is only a test of the sport opportunity perceptions within schools and communities as variables experienced by individual adolescents in those settings. Consequently, if data for schools or communities can be collected in future studies, multilevel modelling approaches could be utilised to test for inequalities within schools or communities.

### 5.5 Equity interpretation for rural and low-SES adolescents

These findings regarding rural residential areas are especially important because they show that disadvantage cannot be reduced to the socioeconomic status of individuals in those households alone. Rural adolescents had fewer opportunities to participate in school- and community-based sports, and also exhibited lower mental wellbeing after adjusting for various covariates. These outcomes are in part in alignment with research that indicates that rural schools often have a lack of physical education teachers for those schools (29), and national evidence also shows persistent rural–urban differences in physical fitness among Chinese children and adolescents (30).

While the disadvantages for adolescents of low socioeconomic status living in urban areas may differ from those in rural areas, they are similarly restrictive in their effects on adolescents’ lives. For instance, while sports facilities may be available in urban areas, they may not be accessible to low-SES adolescents due to cost or limited accessibility. Furthermore, clubs in the area may require students and their parents to contribute fees, time, or resources, which may not be available to disadvantaged families. Thus, disadvantages in these areas can reduce adolescents’ self-efficacy to participate in physical activity.

### 5.6 Strengths and limitations

The study has several strengths. Firstly, the three-wave longitudinal structure of the study allows the model’s constructs to be ordered over time. Secondly, the model includes both school-based and community-based sport opportunity variables, allowing for comparison between them. Thirdly, there were adjustments for baseline levels of mental wellbeing, age, sex, rural residence, and grade level. Fourthly, the use of full-information maximum likelihood estimation helps account for information lost due to incomplete follow-up of participants. Finally, bootstrap confidence intervals indicate that the model provides a robust test of the indirect effects.

Several limitations to the study should also be discussed. First, and most importantly, the Time 2 constructs were measured within the same wave, so their hypothesised sequence is theoretically motivated rather than directly observed and the design cannot rule out alternative or reciprocal orderings (Section 3.4). Second, the measures were developed for this study without formal validation beyond exploratory factor analysis, and the SES composite combines formative indicators (Sections 3.2 and 3.3). Third, individual school identifiers were not available, so school-level clustering could not be quantified or adjusted for and standard errors may be underestimated. Fourth, the sample, although spanning 12 provinces and municipalities, was a multi-site convenience sample, so findings should be generalised to Chinese adolescents with caution. Fifth, exercise self-efficacy was measured as barrier self-efficacy, and no direct path from self-efficacy to mental wellbeing was estimated (Section 5.3). Sixth, the data were self-reported. Seventh, the study did not model the measurement error inherent to composite scores. Eighth, the sample size may be insufficient for more complex models. Ninth, attrition in the follow-up surveys reduced the sample to 240 adolescents with complete data; retained and non-retained participants did not differ significantly on baseline characteristics (Section 4.1a), which lends some support to a missing-at-random mechanism, although attrition related to unmeasured variables cannot be ruled out. Finally, the TLI and RMSEA indicated poor absolute fit (Sections 4.2 and 5.4), so the model should be regarded as provisional.

## 6 Policy and practice implications

Because this study is observational and cross-sectional in its measurement of the Time 2 mediators, the findings below identify potentially modifiable correlates and plausible targets for future intervention research rather than causally established levers for change; they should inform, rather than substitute for, further intervention studies before being translated into policy. The findings have some implications regarding reducing mental health inequalities among adolescents in China. One implication of the study is that increasing access to school-based sports could improve adolescents’ mental wellbeing. Schools in disadvantaged areas of China should be provided with minimum standards for sports facilities, equipment, and teachers, ensuring that all adolescents have access to at least some opportunities for physical activity. While these benefits are associated with physical health, they may also enhance adolescent students’ mental wellbeing.

Another implication of the study is regarding community sports facilities. While many adolescents in rural and low-income urban areas may not have access to schools with adequate facilities and teachers, subsidising sports programmes in these communities may improve residents’ mental wellbeing. These opportunities for activity outside of school may be especially important for students from schools without sports facilities. Furthermore, because access to community sports programmes was significantly associated with exercise self-efficacy and physical activity levels, the inclusion of such facilities in local governments’ plans may improve adolescents’ mental wellbeing.

Another implication of this research’s findings is the need for physical education classes to build students’ confidence in exercising despite barriers. Because the construct measured here is barrier self-efficacy rather than task self-efficacy, relevant strategies include helping students plan around common obstacles such as tiredness, limited time, or lack of a companion, and teaching simple coping and self-regulation skills. Increasing barrier self-efficacy would therefore mean strengthening motivation and planning to sustain activity, not only improving skills.

The fourth implication of the research is that mental health and physical activity should be promoted together. The association between physical activity and mental wellbeing later in adolescence was the strongest implication of this research. Therefore, schools should integrate physical education, mental health, and health education classes as well as clubs to improve the mental wellbeing of students with mental health issues.

Finally, it would be beneficial for the Chinese government to implement nationwide monitoring systems to track adolescents’ mental wellbeing across different socioeconomic statuses (SES). The Healthy China 2030, the National Fitness Program, the Double Reduction Policy, and various plans regarding student mental health across the nation have already recognised the importance of both the physical and mental health of adolescents. However, these plans can be further developed to specifically address mental health inequalities and monitoring of specific groups of adolescents according to their SES.

## 7 Conclusion

While sports opportunities are not the only factor contributing to adolescents’ mental wellbeing in China, they are one factor that contributes to socioeconomic differences in mental wellbeing. Furthermore, while it is true that factors such as socioeconomic status and rural residence are directly associated with adolescents’ mental wellbeing, the inclusion of sport opportunities as model variables indicates potential targets for intervention to improve mental health. By increasing access to sports opportunities, improving sport infrastructure in Chinese communities, and enhancing adolescents’ mental wellbeing by increasing their self-efficacy in meeting physical activity goals, it may be possible to simultaneously address physical inactivity and mental wellbeing among adolescents with disadvantaged socioeconomic status. Thus, addressing the inequalities in sport opportunities in China is not just an issue of physical fitness but also of mental health.

## Data Availability

The de-identified datasets supporting the findings of this study are available from the corresponding author upon reasonable request, subject to applicable ethical and privacy restrictions. Requests for data access will be reviewed to ensure compliance with the informed consent procedures and the conditions of ethical approval, particularly regarding the protection of adolescent participants’ confidentiality.

## References

[1] World Health Organization. Adolescent mental health (2021). Available online at: https://www.who.int/news-room/fact-sheets/detail/adolescent-mental-health (Accessed October 9, 2026).

[2] Fu X Zhang K Chen X. Mental Health Blue Book: Chinese National Mental Health Development Report 2019–2020. Beijing, China: Social Science Academic Press (2021).

[3] Zhou J Liu Y Ma J Feng Z Hu J Hu J . Prevalence of depressive symptoms among children and adolescents in China: a systematic review and meta-analysis. Child Adolesc Psychiatry Ment Health. (2024) 18:150. doi: 10.1186/s13034-024-00841-w, 39563377 PMC11577650

[4] Tang S Xiang M Cheung T Xiang YT. Mental health and its correlates among children and adolescents during COVID-19 school closure: the importance of parent-child discussion. J Affect Disord. (2021) 279:353–60. doi: 10.1016/j.jad.2020.10.016, 33099049 PMC7550131

[5] Liu J Zeng M Wang D Zhang Y Shang B Ma X. Applying social cognitive theory in predicting physical activity among Chinese adolescents: a cross-sectional study with multigroup structural equation model. Front Psychol. (2022) 12:695241. doi: 10.3389/fpsyg.2021.695241, 35370866 PMC8965556

[6] National Bureau of Statistics of China. China Statistical Yearbook 2023. Beijing, China: China Statistics Press (2023).

[7] Xie Y Zhou X. Income inequality in today's China. Proc Natl Acad Sci USA. (2014) 111:6928–33. doi: 10.1073/pnas.1403158111, 24778237 PMC4024912

[8] Recchia F Bernal JDK Fong DY Wong SHS Chung P-K Chan DKC . Physical activity interventions to alleviate depressive symptoms in children and adolescents: a systematic review and meta-analysis. JAMA Pediatr. (2023) 177:132–40. doi: 10.1001/jamapediatrics.2022.5090, 36595284 PMC9857695

[9] Ke Y Shi L Peng L Chen S Hong J Liu Y. Associations between socioeconomic status and physical activity: a cross-sectional analysis of Chinese children and adolescents. Front Psychol. (2022) 13:904506. doi: 10.3389/fpsyg.2022.904506, 36118481 PMC9477139

[10] Wang J Wu S Chen X Xu B Wang J Yang Y . Impact of awareness of sports policies, school, family, and community environmental on physical activity and fitness among children and adolescents: a structural equation modeling study. BMC Public Health. (2024) 24:2298. doi: 10.1186/s12889-024-19795-x, 39256716 PMC11389504

[11] Beauchamp MR Crawford KL Jackson B. Social cognitive theory and physical activity: mechanisms of behavior change, critique, and legacy. Psychol Sport Exerc. (2019) 42:110–7. doi: 10.1016/j.psychsport.2018.11.009

[12] Egele VS Klopp E Stark R. How valid is bandura's social cognitive theory to explain physical activity behavior? Eur J Investig Health Psychol Educ. (2025) 15:20. doi: 10.3390/ejihpe15020020, 39997084 PMC11854433

[13] Du H Chi P King RB. Economic inequality is associated with long-term harm on adolescent well-being in China. Child Dev. (2019) 90:1016–1026. doi: 10.1111/cdev.13253 31074053

[14] Wang X Wang Y Zhang X Yang W Yang J. A large-scale cross-sectional study on mental health status among children and adolescents: Jiangsu Province, China, 2022. China CDC Weekly. (2023) 5:710–4. doi: 10.46234/ccdcw2023.136, 37614908 PMC10442699

[15] Yang D Hu S Li M. The influence of family socioeconomic status on adolescents' mental health in China. Int J Environ Res Public Health. (2022) 19:Article 7824. doi: 10.3390/ijerph19137824, 35805483 PMC9265672

[16] Zhang X Yang H Zhang J Yang M Yuan N Liu J. Prevalence of and risk factors for depressive and anxiety symptoms in a large sample of Chinese adolescents in the post-COVID-19 era. Child Adolesc Psychiatry Ment Health. (2021) 15:80. doi: 10.1186/s13034-021-00429-8, 34961519 PMC8710930

[17] Reiss F Erhart M Kaman A Devine J Zoellner F Ravens-Sieberer U. Tracing socioeconomic inequalities in children's and adolescents' mental health: longitudinal study findings from 2017 to 2024. BMC Public Health. (2026) 26:711. doi: 10.1186/s12889-026-26577-0, 41703520 PMC12930680

[18] Zheng N. The relationship between socioeconomic status and organized sports participation among Chinese children and adolescents: the chain-mediated role of parental physical exercise and parental support. BMC Public Health. (2025) 25:1742. doi: 10.1186/s12889-025-23187-0, 40420302 PMC12105234

[19] Li Z Xie S Chen W. The influence of environment on adolescents' physical exercise behavior based on family, community, and school micro-systems. Sci Rep. (2025) 15:11849. doi: 10.1038/s41598-025-91387-0, 40199914 PMC11978958

[20] Peng B Chen W Wang H Yu T. How does physical exercise influence self-efficacy in adolescents? A study based on the mediating role of psychological resilience. BMC Psychol. (2025) 13:285. doi: 10.1186/s40359-025-02529-y, 40119462 PMC11927186

[21] Ren Z Hu L Yu JJ Yu Q Chen S Ma Y . The influence of social support on physical activity in Chinese adolescents: the mediating role of exercise self-efficacy. Children. (2020) 7:23. doi: 10.3390/children7030023, 32245103 PMC7140834

[22] Xu Z Xu J Liu T Gu Z Hu D. The pathway of social support in enhancing adolescents' physical fitness: the mediating roles of physical activity and self-efficacy. PLoS One. (2024) 19:e0308864. doi: 10.1371/journal.pone.0308864, 39325714 PMC11426479

[23] Wu J Shao Y Zang W Hu J. Is physical exercise associated with reduced adolescent social anxiety mediated by psychological resilience? Evidence from a longitudinal multi-wave study in China. Child Adolesc Psychiatry Ment Health. (2025) 19:17. doi: 10.1186/s13034-025-00867-8, 40045423 PMC11884043

[24] Li Q Li L Li C Wang H. The association between moderate-to-vigorous physical activity and health-related quality of life in Chinese adolescents: the mediating roles of emotional intelligence and perceived stress. Front Psychol. (2024) 15:1477018. doi: 10.3389/fpsyg.2024.1477018, 39687563 PMC11646723

[25] Zhang G Feng W Zhao L Zhao X Li T. The association between physical activity, self-efficacy, stress self-management and mental health among adolescents. Sci Rep. (2024) 14:5488. doi: 10.1038/s41598-024-56149-4, 38448518 PMC10917799

[26] Zhang M Gu C Zeng H Yi X. The impact of physical activity on adolescent mental health status: the mediating effect of school adaptation. Front Psychol. (2025) 16:1573129. doi: 10.3389/fpsyg.2025.1573129, 40443729 PMC12119619

[27] Guthold R Stevens GA Riley LM Bull FC. Global trends in insufficient physical activity among adolescents: a pooled analysis of 298 population-based surveys with 1.6 million participants. Lancet Child Adolesc Health. (2020) 4:23–35. doi: 10.1016/S2352-4642(19)30323-2, 31761562 PMC6919336

[28] Liu Y Ke Y Liang Y Zhu Z Cao Z Zhuang J . Results from the China 2022 report card on physical activity for children and adolescents. J Exerc Sci Fit. (2023) 21:1–5. doi: 10.1016/j.jesf.2022.10.004, 36349305 PMC9634471

[29] Wang J Cui Y Liu Y. Effects of school physical education on the exercise habits of children and adolescents: an empirical analysis using China health and nutrition survey data. J Sch Health. (2024) 94:23–36. doi: 10.1111/josh.13391, 37697677

[30] Cai S Li J Huang T Liu Y Dang J Chen Z . Changing landscape of physical fitness and urban-rural inequalities: evidence from 1.2 million children and adolescents over 20 years of urbanization. BMC Med. (2026) 24:175. doi: 10.1186/s12916-026-04712-3, 41715134 PMC13023139

[31] R Core Team. R: A Language and Environment for Statistical Computing. Vienna, Austria: R Foundation for Statistical Computing (2024).

[32] Rosseel Y. Lavaan: an R package for structural equation modeling. J Stat Softw. (2012) 48:1–36. doi: 10.18637/jss.v048.i02

