# Supplementary material for: Socioeconomic inequalities in adolescent mental wellbeing in China—a longitudinal path analysis of sport opportunities, exercise self-efficacy, and physical activity
Source: Front Public Health. 2026 Oct 20;14:1916205. doi: 10.3389/fpubh.2026.1916205 (PMC13727467; doi:10.3389/fpubh.2026.1916205)
Supplement: Supplementary file 1 [file Supplementary_file_1.docx]

# Supplementary File S1

## Supplementary Statistical Analyses

*Manuscript. Socioeconomic Inequalities in Adolescent Mental Well-Being in China - A Longitudinal Path Analysis of Sport Opportunities, Exercise Self-Efficacy, and Physical Activity*

This file reports the complete statistical output underlying the path analysis summarised in Tables 2-4 of the main manuscript, including the full structural regression results, residual and exogenous covariances, R-square values, bootstrapped indirect and total effects with 95% confidence intervals, and model fit indices. All values are taken directly from the fitted lavaan model and are reported to three decimal places as produced by the software.

## S1.1 Analytic Approach

The hypothesised path model was fitted in R (version 4.x; R Core Team, 2024) using the lavaan package (version 0.6-21; Rosseel, 2012). The model was estimated with maximum likelihood (ML), using full-information maximum likelihood (FIML) to handle missing data under all 328 Time 1 participants. Standard errors and confidence intervals for all parameters were obtained using the non-parametric bootstrap (1,000 requested draws; 1,000 successful draws). The model included 74 estimated parameters.

## S1.2 Model Fit Indices

| **Index** | **Value** |
| --- | --- |
| Number of observations | 328 |
| Number of missing data patterns | 3 |
| Model test statistic (chi-square) | 36.980 |
| Degrees of freedom | 3 |
| P-value (chi-square) | < .001 |
| Baseline model chi-square | 1484.445 |
| Baseline model degrees of freedom | 40 |
| Comparative Fit Index (CFI) | 0.976 |
| Tucker-Lewis Index (TLI) | 0.686 |
| Robust CFI | 0.971 |
| Robust TLI | 0.619 |
| RMSEA | 0.186 |
| RMSEA 90% CI | [0.135, 0.242] |
| P-value, H0: RMSEA <= .050 | < .001 |
| P-value, H0: RMSEA >= .080 | 1.000 |
| Robust RMSEA | 0.220 |
| Robust RMSEA 90% CI | [0.160, 0.286] |
| SRMR | 0.014 |
| Loglikelihood, fitted model (H0) | -3490.934 |
| Loglikelihood, unrestricted model (H1) | -3472.444 |
| Akaike Information Criterion (AIC) | 7129.869 |
| Bayesian Information Criterion (BIC) | 7410.552 |
| Sample-size adjusted BIC (SABIC) | 7175.826 |

Note. The robust CFI, TLI, and RMSEA are reported as produced by lavaan alongside the standard (non-robust) indices; the manuscript interprets the standard ML-based indices shown in Table 2, consistent with the ML estimator used to fit the model. Given the model’s very low degrees of freedom (df = 3), RMSEA and TLI should be interpreted cautiously, as both indices are known to behave unpredictably in low-df models (see Sections 4.2 and 5.4 of the manuscript).

## S1.3 Full Structural Regression Results

Unstandardised estimates, bootstrap standard errors, z-values, p-values, 95% bootstrap confidence intervals, and standardised (Std.all) coefficients for every estimated structural path, including all covariates. Path labels in parentheses (e.g., a1, a2) correspond to the labels used to define the indirect and total effects in Section S1.6.

| **Outcome** | **Predictor** | **Est.** | **SE** | **z** | **p** | **95% CI** | **Std.all** |
| --- | --- | --- | --- | --- | --- | --- | --- |
| School sport opportunities T2 | SES T1 (a1) | 0.493 | 0.048 | 10.223 | < .001 | [0.401, 0.591] | .443 |
|  | Baseline mental well-being | 0.207 | 0.046 | 4.495 | < .001 | [0.109, 0.291] | .199 |
|  | Age | -0.039 | 0.054 | -0.719 | .472 | [-0.150, 0.067] | -.049 |
|  | Sex (female) | 0.051 | 0.088 | 0.574 | .566 | [-0.121, 0.213] | .022 |
|  | Rural residence | -0.903 | 0.102 | -8.880 | < .001 | [-1.103, -0.699] | -.377 |
|  | Senior grade | 0.252 | 0.165 | 1.527 | .127 | [-0.082, 0.566] | .107 |
| Community sport opportunities T2 | SES T1 (a2) | 0.506 | 0.054 | 9.400 | < .001 | [0.402, 0.617] | .457 |
|  | Baseline mental well-being | 0.062 | 0.046 | 1.349 | .177 | [-0.028, 0.152] | .060 |
|  | Age | -0.072 | 0.059 | -1.236 | .216 | [-0.199, 0.036] | -.092 |
|  | Sex (female) | 0.023 | 0.092 | 0.250 | .802 | [-0.155, 0.200] | .010 |
|  | Rural residence | -0.971 | 0.109 | -8.892 | < .001 | [-1.168, -0.747] | -.408 |
|  | Senior grade | 0.093 | 0.180 | 0.518 | .604 | [-0.276, 0.457] | .040 |
| Exercise self-efficacy T2 | School sport opportunities T2 (b1) | 0.321 | 0.061 | 5.266 | < .001 | [0.199, 0.440] | .327 |
|  | Community sport opportunities T2 (b2) | 0.334 | 0.056 | 5.939 | < .001 | [0.219, 0.440] | .339 |
|  | SES T1 | 0.144 | 0.064 | 2.247 | .025 | [0.022, 0.270] | .132 |
|  | Baseline mental well-being | 0.087 | 0.051 | 1.721 | .085 | [-0.008, 0.190] | .086 |
|  | Age | -0.015 | 0.054 | -0.275 | .783 | [-0.122, 0.089] | -.019 |
|  | Sex (female) | 0.031 | 0.092 | 0.342 | .733 | [-0.157, 0.218] | .014 |
|  | Rural residence | -0.091 | 0.125 | -0.724 | .469 | [-0.337, 0.160] | -.039 |
|  | Senior grade | 0.030 | 0.167 | 0.179 | .858 | [-0.299, 0.365] | .013 |
| Physical activity T2 | Exercise self-efficacy T2 (c1) | 0.328 | 0.056 | 5.858 | < .001 | [0.217, 0.442] | .354 |
|  | School sport opportunities T2 (c2) | 0.244 | 0.052 | 4.708 | < .001 | [0.146, 0.351] | .268 |
|  | Community sport opportunities T2 (c3) | 0.276 | 0.052 | 5.352 | < .001 | [0.175, 0.376] | .301 |
|  | SES T1 | -0.040 | 0.056 | -0.721 | .471 | [-0.143, 0.071] | -.040 |
|  | Baseline mental well-being | 0.116 | 0.042 | 2.766 | .006 | [0.033, 0.195] | .123 |
|  | Age | -0.021 | 0.049 | -0.437 | .662 | [-0.116, 0.074] | -.030 |
|  | Sex (female) | -0.003 | 0.071 | -0.048 | .962 | [-0.151, 0.136] | -.002 |
|  | Rural residence | 0.149 | 0.105 | 1.424 | .154 | [-0.045, 0.370] | .069 |
|  | Senior grade | 0.115 | 0.148 | 0.783 | .434 | [-0.174, 0.408] | .054 |
| Mental well-being T3 | Physical activity T2 (d1) | 0.567 | 0.051 | 11.177 | < .001 | [0.471, 0.664] | .536 |
|  | SES T1 (cp) | 0.188 | 0.048 | 3.915 | < .001 | [0.091, 0.281] | .176 |
|  | Baseline mental well-being | 0.249 | 0.045 | 5.559 | < .001 | [0.159, 0.338] | .249 |
|  | Age | 0.070 | 0.048 | 1.444 | .149 | [-0.024, 0.165] | .091 |
|  | Sex (female) | -0.072 | 0.070 | -1.033 | .302 | [-0.219, 0.052] | -.032 |
|  | Rural residence | -0.287 | 0.095 | -3.015 | .003 | [-0.483, -0.111] | -.125 |
|  | Senior grade | -0.269 | 0.139 | -1.941 | .052 | [-0.539, -0.017] | -.119 |

## S1.4 Covariances

| **Pair** | **Est.** | **SE** | **z** | **p** | **95% CI** | **Std.all** |
| --- | --- | --- | --- | --- | --- | --- |
| School sport T2 -- Community sport T2 | 0.048 | 0.039 | 1.225 | .220 | [-0.028, 0.127] | .083 |
| SES T1 -- Baseline mental well-being | 0.550 | 0.067 | 8.190 | < .001 | [0.417, 0.682] | .461 |
| SES T1 -- Age | 0.068 | 0.086 | 0.787 | .431 | [-0.102, 0.227] | .044 |
| SES T1 -- Sex (female) | 0.022 | 0.029 | 0.744 | .457 | [-0.035, 0.079] | .041 |
| SES T1 -- Rural residence | -0.159 | 0.027 | -5.842 | < .001 | [-0.212, -0.104] | -.307 |
| SES T1 -- Senior grade | 0.021 | 0.029 | 0.708 | .479 | [-0.037, 0.076] | .039 |
| Baseline MWB -- Age | -0.018 | 0.095 | -0.188 | .851 | [-0.209, 0.162] | -.011 |
| Baseline MWB -- Sex (female) | -0.048 | 0.031 | -1.545 | .122 | [-0.108, 0.015] | -.085 |
| Baseline MWB -- Rural residence | -0.164 | 0.029 | -5.650 | < .001 | [-0.221, -0.113] | -.296 |
| Baseline MWB -- Senior grade | 0.005 | 0.031 | 0.162 | .871 | [-0.057, 0.069] | .009 |
| Age -- Sex (female) | -0.017 | 0.040 | -0.427 | .670 | [-0.097, 0.059] | -.023 |
| Age -- Rural residence | -0.004 | 0.041 | -0.108 | .914 | [-0.085, 0.077] | -.006 |
| Age -- Senior grade | 0.625 | 0.022 | 28.979 | < .001 | [0.583, 0.665] | .845 |
| Sex (female) -- Rural residence | 0.000 | 0.014 | 0.015 | .988 | [-0.026, 0.029] | .001 |
| Sex (female) -- Senior grade | -0.015 | 0.013 | -1.127 | .260 | [-0.041, 0.011] | -.060 |
| Rural residence -- Senior grade | -0.006 | 0.014 | -0.418 | .676 | [-0.034, 0.020] | -.023 |

Note. The covariance between school and community sport opportunities was freely estimated to account for their shared dependence on common antecedents. Covariances among the exogenous predictors (SES, baseline mental well-being, age, sex, rural residence, grade) are estimated by default in this model specification and are reported here for completeness; they are not substantively interpreted in the manuscript. MWB = mental well-being.

## S1.5 R-Square Values

| **Endogenous variable** | **R-square** |
| --- | --- |
| School sport opportunities T2 | .616 |
| Community sport opportunities T2 | .536 |
| Exercise self-efficacy T2 | .589 |
| Physical activity T2 | .664 |
| Mental well-being T3 | .769 |

## S1.6 Bootstrapped Indirect and Total Effects

Defined parameters below were computed from the path coefficients labelled in Section S1.3 (a1, a2, b1, b2, c1, d1, cp), using 1,000 bootstrap draws (percentile method). All values are unstandardised.

| **Effect** | **Definition** | **Est.** | **SE** | **z** | **p** | **95% CI** | **Std.all** |
| --- | --- | --- | --- | --- | --- | --- | --- |
| Indirect via school sport pathway | a1 × b1 × c1 × d1 | 0.029 | 0.009 | 3.206 | .001 | [0.014, 0.050] | .027 |
| Indirect via community sport pathway | a2 × b2 × c1 × d1 | 0.031 | 0.009 | 3.311 | .001 | [0.016, 0.053] | .029 |
| Total indirect effect | sum of the two pathways above | 0.061 | 0.016 | 3.795 | < .001 | [0.033, 0.096] | .057 |
| Total effect of SES on MWB T3 | cp + total indirect effect | 0.249 | 0.050 | 5.032 | < .001 | [0.152, 0.348] | .233 |

Note. "Indirect via school sport pathway" and "via community sport pathway" correspond to ind_school_seq and ind_community_seq in the lavaan output, respectively, and are reported in the manuscript (Table 4; Section 4.6) as the indirect effects of socioeconomic status on Time 3 mental well-being operating sequentially through school and community sport opportunities, exercise self-efficacy, and physical activity. Both routes share the c1 (self-efficacy to physical activity) and d1 (physical activity to mental well-being) legs of the pathway; they differ only in whether sport opportunities are accessed through school (a1, b1) or through the community (a2, b2). MWB = mental well-being.

## S1.7 Model Syntax

The lavaan model syntax used to produce the results reported above and in the main manuscript is as follows.

```
model <- '
  # Structural paths
  SchoolSport_T2    - a1*SES_T1 + MWB_T1 + Age + Gender_Female + Residence_Rural + Grade_Senior
  CommunitySport_T2 - a2*SES_T1 + MWB_T1 + Age + Gender_Female + Residence_Rural + Grade_Senior
  SelfEfficacy_T2   - b1*SchoolSport_T2 + b2*CommunitySport_T2 + SES_T1 + MWB_T1 +
                       Age + Gender_Female + Residence_Rural + Grade_Senior
  PA_T2             - c1*SelfEfficacy_T2 + c2*SchoolSport_T2 + c3*CommunitySport_T2 +
                       SES_T1 + MWB_T1 + Age + Gender_Female + Residence_Rural + Grade_Senior
  MWB_T3            - d1*PA_T2 + cp*SES_T1 + MWB_T1 + Age + Gender_Female +
                       Residence_Rural + Grade_Senior

  # Residual covariance
  SchoolSport_T2 -- CommunitySport_T2

  # Defined parameters
  ind_school_seq    := a1*b1*c1*d1
  ind_community_seq := a2*b2*c1*d1
  total_indirect    := ind_school_seq + ind_community_seq
  total_effect      := cp + total_indirect
'

fit <- sem(model, data = wide, missing = "fiml", estimator = "ML",
           se = "bootstrap", bootstrap = 1000, fixed.x = FALSE)
```

Note. SES T1 is included as a covariate-level predictor of exercise self-efficacy (direct path) in addition to its role as the originating variable of the sequential indirect pathways; this direct path (β = .132, p = .025) is reported in Table 3 of the main manuscript and discussed in Section 5.2.
