# Supplementary material for: Socioeconomic inequalities in adolescent mental wellbeing in China—a longitudinal path analysis of sport opportunities, exercise self-efficacy, and physical activity
Source: Front Public Health. 2026 Oct 20;14:1916205. doi: 10.3389/fpubh.2026.1916205 (PMC13727467; doi:10.3389/fpubh.2026.1916205)
Supplement: Supplementary file 2 [file Supplementary_file_2.docx]

# Supplementary File S2

## Questionnaire and Measurement Items

*Manuscript. Socioeconomic Inequalities in Adolescent Mental Well-Being in China - A Longitudinal Path Analysis of Sport Opportunities, Exercise Self-Efficacy, and Physical Activity*

This file reproduces the complete original questionnaire administered to participants, including all items, response options, measurement scales, and scoring procedures used to derive the composite variables analysed in the main manuscript (SES_T1, SchoolSport_T2, CommunitySport_T2, SelfEfficacy_T2, PA_T2, MWB_T1, MWB_T3). Item numbering (1-70) follows the original instrument. Section letters (A-H) refer to the instrument's own section headings.

Instructions to participants (as given on the instrument). This questionnaire asks about your family background, school and community sport opportunities, confidence in exercise, physical activity, and mental well-being. There are no right or wrong answers. Please answer honestly. Your responses will be kept confidential and used only for research purposes.

## S2.1 Overview of Questionnaire Structure and Wave Timing

The questionnaire was administered across three waves. Section B (socioeconomic status) was collected once, at Time 1, and the same values were carried forward to represent SES at later waves; this was confirmed empirically by comparing values for the same participant IDs across wave-sheets of the raw data (100% identical values for SES items between Time 1 and Time 2 records). Sections C-F (school sport, community sport, self-efficacy, physical activity) were genuinely re-measured at Time 2 (partial, non-identical overlap with other waves in the raw data). Section G (mental well-being) used the same 15-item instrument at both Time 1 (baseline) and Time 3 (outcome). Section H (support) was collected at Time 2 but was not used to construct any variable in the present path model.

| **Wave** | **Sections collected** | **Variables derived** |
| --- | --- | --- |
| Time 1 (n = 328) | A (background), B (SES), G (mental well-being, baseline) | SES_T1, MWB_T1, Age, Gender_Female, Residence_Rural, Grade_Senior, School_Private, Lives_Not_Both_Parents |
| Time 2 (n = 294) | C (school sport), D (community sport), E (self-efficacy), F (physical activity), H (support, not modelled) | SchoolSport_T2, CommunitySport_T2, SelfEfficacy_T2, PA_T2 |
| Time 3 (n = 240) | G (mental well-being, outcome) | MWB_T3 |

## S2.2 Section A: Background Information (Time 1, Items 1-7)

| **Item** | **Statement** |
| --- | --- |
| 1 | Age: ____________________ (open response, years) |
| 2 | Gender: [ ] Male [ ] Female [ ] Prefer not to say |
| 3 | Grade level: [ ] Junior secondary [ ] Senior secondary |
| 4 | Place of residence: [ ] Urban [ ] Rural |
| 5 | Province/region: ____________________ (open response) |
| 6 | Type of school: [ ] Public school [ ] Private school |
| 7 | Do you live with both parents? [ ] Yes [ ] No |

*Scoring. Age and Province/Region were retained as reported. Gender, Grade level, Residence, School type, and Lives with both parents were each recoded as the binary covariates Gender_Female, Grade_Senior, Residence_Rural, School_Private, and Lives_Not_Both_Parents (1 = category named, 0 = otherwise) for use as covariates in the path model.*

## S2.3 Section B: Socioeconomic Status (Time 1, Items 8-13)

| **Item** | **Statement** |
| --- | --- |
| 8 | What is your father's highest level of education? [ ] Primary school or below [ ] Junior secondary school [ ] Senior secondary school [ ] College/diploma [ ] University or above |
| 9 | What is your mother's highest level of education? [ ] Primary school or below [ ] Junior secondary school [ ] Senior secondary school [ ] College/diploma [ ] University or above |
| 10 | How would you describe your family's economic situation? [ ] Very poor [ ] Poor [ ] Average [ ] Good [ ] Very good |
| 11 | Does your family usually have enough money to pay for your school needs? [ ] Never [ ] Rarely [ ] Sometimes [ ] Often [ ] Always |
| 12 | Does your family usually have enough money to pay for sport or exercise activities? [ ] Never [ ] Rarely [ ] Sometimes [ ] Often [ ] Always |
| 13 | Which of the following does your household have? Tick all that apply. [ ] Smartphone [ ] Computer/laptop [ ] Internet access [ ] Private study room [ ] Bicycle [ ] Car [ ] Sports equipment |

*Scoring. Items 8 and 9 were each coded 1-5 ordinally (Primary school or below = 1 ... University or above = 5). Items 10-12 were each coded 1-5 ordinally in the order shown. Item 13 was coded as seven separate binary indicators (1 = household has the item, 0 = does not) and rescaled to a 0-1 index (number of items present / 7). The composite SES_T1 score was computed as the mean of the five 1-5 items (8, 9, 10, 11, 12) together with the rescaled household-asset index from item 13, after standardising each component.*

## S2.4 Section C: School Sport Opportunities (Time 2, Items 14-23)

| **Item** | **Statement** |
| --- | --- |
| 14 | My school has enough sports facilities for students. |
| 15 | My school has enough sports equipment. |
| 16 | My school provides regular physical education lessons. |
| 17 | My school encourages students to participate in sport. |
| 18 | My school offers extracurricular sport clubs or teams. |
| 19 | Teachers support students who want to be physically active. |
| 20 | Students from all family backgrounds can participate in school sports. |
| 21 | School sport activities are affordable for most students. |
| 22 | I feel safe using school sport facilities. |
| 23 | My school gives girls and boys equal chances to participate in sport. |

*Response scale. 1 = Strongly disagree; 2 = Disagree; 3 = Not sure; 4 = Agree; 5 = Strongly agree. Scoring. SchoolSport_T2 was computed as the mean of items 14-23.*

## S2.5 Section D: Community Sport Opportunities (Time 2, Items 24-33)

| **Item** | **Statement** |
| --- | --- |
| 24 | There are sports facilities near my home. |
| 25 | Parks, courts, playgrounds, or exercise areas are easy to reach from my home. |
| 26 | Community sport facilities are safe to use. |
| 27 | Community sport facilities are affordable for my family. |
| 28 | There are organised sport activities for adolescents in my community. |
| 29 | My community has enough open spaces for exercise. |
| 30 | I can access community sport facilities without difficulty. |
| 31 | My parents/guardians allow me to use community sport spaces. |
| 32 | My neighbourhood is safe for walking, running, or playing sports. |
| 33 | Low-income families in my community can still access sport activities. |

*Response scale. 1 = Strongly disagree; 2 = Disagree; 3 = Not sure; 4 = Agree; 5 = Strongly agree. Scoring. CommunitySport_T2 was computed as the mean of items 24-33.*

## S2.6 Section E: Exercise Self-Efficacy (Time 2, Items 34-41)

| **Item** | **Statement** |
| --- | --- |
| 34 | I am confident that I can exercise even when I feel tired. |
| 35 | I am confident that I can exercise even when I have schoolwork. |
| 36 | I am confident that I can exercise even when my friends do not join me. |
| 37 | I am confident that I can exercise regularly every week. |
| 38 | I am confident that I can learn new sports or physical activities. |
| 39 | I am confident that I can continue exercising even if I am not very good at it. |
| 40 | I am confident that I can find time for physical activity. |
| 41 | I am confident that I can be physically active even without expensive equipment. |

*Response scale. 1 = Not confident at all; 2 = Slightly confident; 3 = Moderately confident; 4 = Confident; 5 = Very confident. Scoring. SelfEfficacy_T2 was computed as the mean of items 34-41. All eight items ask about confidence in overcoming specific situational barriers to exercise (tiredness, competing schoolwork, lack of company, cost, skill level) rather than confidence in exercise performance or technique, so this measure is best characterised as barrier self-efficacy rather than task self-efficacy.*

## S2.7 Section F: Physical Activity (Time 2, Items 42-48)

| **Item** | **Statement** |
| --- | --- |
| 42 | During the past 7 days, on how many days were you physically active for at least 60 minutes? [ ] 0 days ... [ ] 7 days |
| 43 | How often do you take part in school physical education lessons? [ ] Never [ ] Rarely [ ] Sometimes [ ] Often [ ] Always |
| 44 | How often do you participate in sport after school? [ ] Never [ ] Rarely [ ] Sometimes [ ] Often [ ] Always |
| 45 | How often do you exercise during weekends? [ ] Never [ ] Rarely [ ] Sometimes [ ] Often [ ] Always |
| 46 | How often do you participate in organised sport clubs or teams? [ ] Never [ ] Rarely [ ] Sometimes [ ] Often [ ] Always |
| 47 | What physical activities do you usually do? Tick all that apply. [ ] Running/jogging [ ] Walking [ ] Basketball [ ] Football [ ] Table tennis [ ] Badminton [ ] Swimming [ ] Cycling [ ] Dance [ ] Martial arts [ ] Other: ____________ |
| 48 | On a normal school day, how much time do you spend sitting outside class time, such as using a phone, watching videos, or playing games? [ ] Less than 1 hour [ ] 1-2 hours [ ] 3-4 hours [ ] 5-6 hours [ ] More than 6 hours |

*Scoring. PA_T2 was computed as the mean of items 42-46 (the 0-7 day count in item 42 and the four 1-5 frequency items in items 43-46). PA_T2 is therefore a composite frequency index across activity settings and does not represent minutes of moderate-to-vigorous physical activity directly. Items 47 (activity type) and 48 (sedentary time) were collected for descriptive purposes and were not used to construct any variable in the present path model.*

## S2.8 Section G: Mental Well-Being (Time 1 and Time 3, Items 49-63)

| **Item** | **Statement** |
| --- | --- |
| 49 | I feel cheerful and in good spirits. |
| 50 | I feel calm and relaxed. |
| 51 | I feel active and energetic. |
| 52 | I wake up feeling fresh and rested. |
| 53 | My daily life feels interesting. |
| 54 | I feel hopeful about my future. |
| 55 | I feel satisfied with my life. |
| 56 | I feel supported by people around me. |
| 57 | I can cope with school pressure. |
| 58 | I feel good about myself. |
| 59 | I feel sad or low. (reverse-scored) |
| 60 | I feel nervous or anxious. (reverse-scored) |
| 61 | I feel lonely. (reverse-scored) |
| 62 | I feel stressed because of schoolwork. (reverse-scored) |
| 63 | I find it hard to enjoy things I usually like. (reverse-scored) |

*Response scale. 1 = Never; 2 = Rarely; 3 = Sometimes; 4 = Often; 5 = Always. Scoring. Items 59-63 were reverse-scored (6 - raw response) so that higher values consistently indicate better mental well-being; all 15 items were then averaged. This instrument was administered identically at Time 1 (producing MWB_T1, the baseline covariate) and at Time 3 (producing MWB_T3, the outcome variable).*

## S2.9 Section H: Family, Peer, and School Support (Time 2, Items 64-70)

| **Item** | **Statement** |
| --- | --- |
| 64 | My parents/guardians encourage me to exercise. |
| 65 | My parents/guardians can afford sport-related costs when needed. |
| 66 | My friends encourage me to be physically active. |
| 67 | My teachers encourage me to participate in sport. |
| 68 | I feel connected to my school. |
| 69 | I feel that I belong in my school. |
| 70 | I have someone to talk to when I feel stressed. |

*Response scale. 1 = Strongly disagree; 2 = Disagree; 3 = Not sure; 4 = Agree; 5 = Strongly agree. Scoring. A Support_Score composite (mean of items 64-70) can be derived from these items, but it was not entered into the path model reported in the main manuscript and is included here only for completeness and to support future analyses.*

## S2.10 Derived Composite Scores Used in the Path Model

| **Composite** | **Source items** | **Scale/range** | **Wave** |
| --- | --- | --- | --- |
| SES_T1 | Items 8-13 (5 ordinal items + 7-item asset index) | Standardised composite | Time 1 |
| MWB_T1 | Items 49-63 (10 positive + 5 reverse-scored) | 1-5 (mean) | Time 1 |
| SchoolSport_T2 | Items 14-23 | 1-5 (mean) | Time 2 |
| CommunitySport_T2 | Items 24-33 | 1-5 (mean) | Time 2 |
| SelfEfficacy_T2 | Items 34-41 | 1-5 (mean) | Time 2 |
| PA_T2 | Items 42-46 | Index (mean) | Time 2 |
| MWB_T3 | Items 49-63 (10 positive + 5 reverse-scored) | 1-5 (mean) | Time 3 |
| Support_Score (not modelled) | Items 64-70 | 1-5 (mean) | Time 2 |

## S2.11 Exploratory Factor Analyses of the Composite Measures

Exploratory factor analyses (principal axis factoring, oblique rotation where more than one factor was extracted; factor retention via parallel analysis) were conducted on the item sets underlying each composite measure prior to averaging. All measures showed sampling adequacy well above conventional thresholds (KMO > .90) and were supported by a single dominant factor.

| **Composite** | **KMO** | **Eigenvalue 1** | **Eigenvalue 2** | **Factors retained** | **Loading range** | **% variance (Factor 1)** |
| --- | --- | --- | --- | --- | --- | --- |
| SES_T1 | .92 | 4.35 | 0.61 | 1 | .67-.87 | 72.4% |
| SchoolSport_T2 | .97 | 7.82 | 0.44 | 1 | .86-.89 | 78.2% |
| CommunitySport_T2 | .97 | 7.80 | 0.46 | 1 | .83-.89 | 78.0% |
| SelfEfficacy_T2 | .96 | 6.31 | 0.38 | 1 | .86-.88 | 78.9% |
| PA_T2 | .91 | 4.00 | 0.35 | 1 | .85-.90 | 80.0% |
| MWB_T1 | .99 | 11.48 | 0.52 | 1 | .85-.88 | 76.5% |
| MWB_T3 | .98 | 11.64 | 0.49 | 1 | .83-.90 | 77.6% |

Note. These results are cited in the main manuscript (Section 4.1a) as support for treating each composite as a single factor. MWB_T1 and MWB_T3 were each factor-analysed on the reverse-scored 15-item set (items 49-63). SES_T1 was factor-analysed on the five ordinal items (8-12) together with the household-asset index (item 13); given that these indicators plausibly act as formative rather than reflective indicators of socioeconomic position (i.e., they need not covary strongly with one another to jointly determine SES), the single-factor EFA result is reported for completeness but should be interpreted alongside this conceptual caveat, which is discussed in the manuscript.
